# Notes for Contributors

**DOI:** 10.1155/S1463924681000540

**Published:** 1981


4. The order of selectivity is $ClO_4^- > I^- > SCN^- > NO_3^- > Br^- > ClO_3^- > Cl^- > HSO_3^-$, $CH_3COO^-$, $HCO_3^-$, $H_2PO_4^-$ or $SO_4^{2-}$ for the liquid membrane electrode, and $I^- > Br^- > SCN^- > HSO_3^- > Cl^- > ClO_4^-$, $NO_3^-$, $ClO_3^-$, $CH_3COO^-$, $HCO_3^-$, $H_2PO_4^-$ or $SO_4^{2-}$ for the solid membrane electrode. The life of electrode is $22^{-4}_{+6}$ and $23^{-5}_{+4}$ days for the liquid and solid membrane electrodes respectively, using an ultrasonic wave cleaner. During the continuous monitoring of thiocyanate at 5.8 ppm in actual industrial waste water samples, the maximum permissible concentrations of co-existing chloride and nitrate ions are 2000 ($Cl^-$) and 6 ($NO_3^-$) ppm and 8 ($Cl^-$) and >3000 ($NO_3^-$) ppm for the liquid and solid membrane electrodes respectively.

The effect of temperature, within-day and day-to-day precision, automatic cleaning of electrode, and maintenance of electrode were also evaluated, but the author can find no difference between the liquid and solid membrane electrodes in this respect.

The method of using a thiocyanate ion-selective electrode with liquid membrane recommended here seems to be superior to that of using a cyanide ion-selective electrode for the determination of thiocyanate since the latter has a three stop procedure for a quantitative transformation of thiocyanate into cyanide [4].

It is therefore concluded that for continuous monitoring of thiocyanate in environmental water samples such as natural and waste waters containing less than 2000 ppm chloride and a few ppm nitrate only a liquid membrane electrode gives satisfactory results.

ACKNOWLEDGEMENT

The author is greatly indebted to Professor Kyoji Tôei and Dr Shoji Motomizu of Okayama University for their encouragement, and also to Japan Exlan Co, Ltd for permission to publish this paper.

REFERENCES

[1] Korenaga, T. (1979), *Mikrochimica Acta [Wien]*, II, 455.
[2] Toei, K. and Kawada, K, (1972), *Japan Analyst*, **21**, 1510.
[3] Snell, F.D. and Snell, C.T. (1958), "Colorimetric Methods of Analysis", Vol II, 3rd Edition, Van Nostrand, New York, USA, pp 783.
[4] Nota, G. (1975), *Analytical Chemistry*, **47**, 763.
[5] Ross, J.W. (1976), *Science*, **156**, 1378.

APPENDIX

The chemistry of the spectrophotometric method for the determination of thiocyanate [3] is as follows. A ferric nitrate reagent solution was prepared by dissolving 50 g of the salt in 500 ml of distilled water, adding 25 ml of concentrated nitric acid, and diluting 1 litre with distilled water. To 5 ml of clear filtrate of sample solution, was added 1 ml of the ferric nitrate reagent solution and after 5 min the absorbance of ferric thiocyanate formed at 550 nm was measured using a reagent blank as reference. The concentration of thiocyanate in the sample solution is determined by comparing these results against a calibration curve prepared from a standard sodium thiocyanate solution.

### Notes for Contributors

### Presentation of manuscripts

Manuscripts should be typed (double-spaced) on one side of the paper only and with generous margins. The title should be brief and informative avoiding the word "new" and its synonyms. The full list of authors with their affiliations and full address(es) should appear on the title page. On a separate sheet an abstract of no more than 150 words is required. This should succinctly describe the scope of the contribution and highlight significant findings or innovations. It should be written in a style which can easily be translated into French and German.

*The Concise Oxford Dictionary* and Fowler's *Modern English Usage* (both published by Oxford University Press) should be used as the standard for spelling and grammar. Abbreviations should be limited to those generally recognised, or where a frequently occuring term is abbreviated it should, in the first instance, be explained thus "flow injection analysis (FIA) ..." and the abbreviation used thereafter. Abbreviations, for standard measures and units should follow SI recommendations. There are various publications giving guidance on the use of SI units.

References should be indicated in the text by numerals following the author's name, i.e. Skeggs [6]. On a separate sheet of paper, list all references in numerical order thus:

[6] Skeggs, L.T., *American Journal of Clinical Pathology*, 1959, **28**, 311.

Note that journal titles are given in full. Where there is more than one author, the form Foreman et al. should be used in the text but all authors should be named in the list of references. When reference is made to a chapter in a book the reference should take the following form:

[7] Malmstadt, H.V. in "Topics in Automatic Chemistry" Ed. Stockwell P.B. and Foreman J.K. 1978 Horwood, Chichester, pp. 68-70.

Only work which has been published or has been accepted for publication should be cited. Avoid the citation of documents which are subject to restricted circulation, patent literature, unpublished work and personal communications. The latter can be mentioned in the text in parenthesis.

To illustrate a paper line diagrams are preferred to photographs. Photographs should only be used when they significantly add to the discussion. Diagrams, charts and graphs should be carefully drawn in black ink on stout card or heavy quality tracing paper. Most illustrations are reduced for publication; to allow for this originals should be between 16 and 36 cm wide (the depth must not exceed 50 cm). The lettering of diagrams should be sufficiently clear to withstand reduction. Except in the case of proper names, all lettering should be in lower case print. If photographs are used they must be supplied in the form of clear, unmounted, glossy, black and white prints. "Instant" photographs are not normally acceptable. All illustrations must be identified on the reverse showing the figure number and the author's name.

Each illustration should have a fully explanitory caption. Captions should be typed together on a separate sheet of paper; they must *not* be an inseparable part of the illustration.

